# Hospital Strategy Uptake and Reductions in Unplanned Readmission Rates for Patients with Heart Failure: A Prospective Study

**DOI:** 10.1007/s11606-014-3105-5

**Published:** 2014-12-19

**Authors:** Elizabeth H. Bradley, Heather Sipsma, Leora I. Horwitz, Chima D. Ndumele, Amanda L. Brewster, Leslie A. Curry, Harlan M. Krumholz

**Affiliations:** 1 Department of Health Policy and Management, Yale School of Public Health, 60 College Street, PO Box 208034, New Haven, 06520-8034 CT USA; 2 Robert Wood Johnson Clinical Scholars Program, Department of Medicine, Yale University School of Medicine, New Haven, CT USA; 3 Division of Delivery Science, Department of Population Health, New York University School of Medicine, New York, NY USA; 4 Center for Healthcare Innovation and Delivery Science, New York University Langone Medical Center, New York, NY USA; 5 Section of Cardiovascular Medicine, Department of Medicine, Yale University School of Medicine, New Haven, CT USA; 6 Center for Outcomes Research and Evaluation, Yale-New Haven Hospital, New Haven, CT USA; 7 Division of General Internal Medicine and Clinical Innovation, Department of Medicine, New York University School of Medicine, New York, NY USA

**Keywords:** readmissions, quality improvement, heart failure, discharge

## Abstract

**BACKGROUND:**

Despite recent reductions in national unplanned readmission rates, we have relatively little understanding of which hospital strategies are most associated with changes in risk-standardized readmission rates (RSRR).

**OBJECTIVE:**

We examined associations between the change in hospital 30-day RSRR for patients with heart failure and the uptake of strategies over 12–18 months in a national sample of hospitals.

**DESIGN:**

We conducted a prospective study of hospitals using a Web-based survey at baseline (November 2010–May 2011, *n* = 599, 91.0 % response rate) and 12–18 months later (November 2011–October 2012, *n* = 501, 83.6 % response rate), with RSRR measured at the same time points. The final analytic sample included 478 hospitals.

**PARTICIPANTS:**

The study included hospitals participating in the Hospital-to-Home (H2H) and State Action on Avoidable Rehospitalizations (STAAR) initiatives.

**MAIN MEASURES:**

We examined associations between change in hospital 30-day RSRR for patients with heart failure and the uptake of strategies previously demonstrated to have increased between baseline and follow-up, using unadjusted and adjusted linear regression.

**KEY RESULTS:**

The average number of strategies taken up from baseline to follow-up was 1.6 (SE = 0.06); approximately one-quarter (25.3 %) of hospitals took up at least three new strategies. Hospitals that adopted the strategy of routinely discharging patients with a follow-up appointment already scheduled experienced significant reductions in RSRR (reduction of 0.63 percentage point, *p* value < 0.05). Hospitals that took up three or more strategies had significantly greater reductions in RSRR compared to hospitals that took up only zero to two strategies (reduction of 1.29 versus 0.57 percentage point, *p* value < 0.05). Among the 117 hospitals that took up three or more strategies, 93 unique combinations of strategies were used.

**CONCLUSIONS:**

Although most individual strategies were not associated with RSRR reduction, hospitals that took up any three or more strategies showed significantly greater reduction in RSRR compared to hospitals that took up fewer than three strategies.

**Electronic supplementary material:**

The online version of this article (doi:10.1007/s11606-014-3105-5) contains supplementary material, which is available to authorized users.

## INTRODUCTION

Reducing hospital readmission rates is a national priority. Between 2007 and 2011, nearly one in five hospitalized Medicare beneficiaries were readmitted within 30 days of discharge, at an annual estimated cost of $17 billion per year.^1^ In 2012, the Medicare Hospital Readmissions Reduction Program, which penalized hospitals financially for high readmission rates,^2^ was launched and several national and regional quality collaboratives^3^^–^ 6 emerged to help hospitals reduce readmissions. The most recent Centers for Medicare and Medicaid Services (CMS) data indicate that the all-cause readmission rate of Medicare beneficiaries decreased from an average of 19 % over the 2007–2011 period to 18.4 % in 2012.^7^

Despite this modest success in reducing readmission rates, we have relatively little understanding of how such reductions have occurred. Previous studies^8^^–^ 11 have examined cross-sectional associations between lower readmission rates and specific strategies, such as providing discharge summaries to primary care physicians or making follow-up appointments for patients before they are discharged. A number of multifaceted programs involving patient education, discharge planning, telephone follow-up, and other elements have successfully reduced readmissions in controlled trials,^9^^,^ 12 ^–^ 18 but it is difficult to distinguish which specific program features influenced changes in readmission rates, because most studies tested bundles of interventions. Also, trials took place at a small number of sites under controlled conditions and may lack generalizability. Longitudinal data on a large, national sample of hospitals linking changes in implemented strategies to changes in risk-standardized 30-day readmission rates (RSRR) have not been reported.

Accordingly, we sought to examine associations between changes in hospital strategies and changes in RSRR in a national sample of hospitals. We studied this question among patients hospitalized with heart failure, a group with high RSRRs. Based on prior results,^19^ we knew that the use of nine strategies (Text Box 1) increased significantly between 2010 and 2012 in a national sample of hospitals that participated in the Hospital-to-Home (H2H) and State Action on Avoidable Rehospitalizations (STAAR) initiatives, providing an opportunity for a longitudinal study to test whether adopting these strategies was associated with a change in RSRR. Findings from this study may be useful in identifying approaches that are most strongly associated with reductions in RSRR over time, and thus provide guidance for institutions seeking to improve patient care and control health care costs.

Text Box 1. Nine Hospital Strategies to Reduce Readmissions Whose Prevalence Increased Significantly Between Baseline and Follow-Up

**Table:** 

**Quality Improvement and Performance Monitoring Strategies**
1. Partnering with other hospitals in the local area to reduce readmissions*
2. Tracking the percent of patients who were discharged with a follow-up appointment already scheduled for within 7 days
3. Tracking the proportion of patients readmitted to another hospital
4. Estimating risk of readmission in a formal way and using it to guide clinical care during hospitalization
**Medication Management Strategies**
5. Having electronic medical record or web-based forms in place to facilitate medication reconciliation
6. Using teach-back techniques for patient and family education
**Discharge and Follow-up Strategies**
7. At discharge, providing patients with heart failure (or their caregivers) written action plans for managing changes in condition*
8. Regularly calling patients after discharge to follow up on post-discharge needs or to provide additional education
9. Discharging patients with an outpatient follow-up appointment already scheduled*

*Use of these strategies has been associated with lower RSRR in prior cross-sectional studies^19^

## METHODS

### Study Design and Sample

We conducted a prospective study to examine changes in the prevalence of hospital strategies to reduce hospital readmission rates among patients with heart failure. We contacted all hospitals that by 1 July 2010 had enrolled in either the H2H National Quality Improvement Initiative^3^ or the STAAR Initiative, a state-based collaborative focusing on reducing readmissions of all patients in Massachusetts, Michigan and Washington, funded by the Commonwealth Fund^4^ (*n* = 658). Respondents were instructed to coordinate with other relevant staff to complete a single survey reflecting hospital practices (Online Appendix). Respondents included staff from quality improvement, cardiology, other clinical departments, case management, care coordination, and nonclinical departments. Many respondents reported having more than one role. Of the 658 hospitals, 599 (91.0 %) completed the initial baseline survey, which was conducted between November 2010 and May 2011.

We re-surveyed these hospitals 12–18 months later, between November 2011 and October 2012. Among the 599 baseline respondent hospitals, a total of 501 hospitals (83.6 %) completed the follow-up survey. We found no significant differences between respondent and non-respondent hospitals at the follow-up survey except in ownership type; hospitals that completed the follow-up survey were more likely to be nonprofit than hospitals that only completed the baseline survey (*p* value < 0.01). Because we wanted to compare H2H and STAAR performance improvement, we excluded hospitals that were enrolled in both H2H and STAAR (*n* = 11), for an eligible sample of 490 hospitals. Of these, 12 were eliminated because they were missing CMS data on RSRR.

### Measures

#### Outcome

The primary outcome was a change in RSRR for patients hospitalized with heart failure. RSRR was computed using the same approach as CMS for public reporting of 30-day RSRR.^20^^,^ 21 Rates were derived using years of Medicare data that best coincided with the baseline (July 2010 to June 2011) and follow-up (July 2011 to June 2012) periods. For use in descriptive statistics, we calculated the change in RSRR between baseline and follow-up by subtracting the baseline RSRR from the follow-up RSRR. Negative values thus indicate a decrease in RSRR. In all modeling analyses, we operationalized change in RSRR between baseline and follow-up by modeling follow-up RSRR adjusted for baseline RSRR.^22^^,^ 23

#### Independent Variables

The main independent variables were dummy variables for the uptake of nine different hospital strategies (listed in Text Box 1) to reduce readmissions. Nine strategies were available for us to test in a longitudinal study design, because these strategies had statistically significant increases in use over the study period.^19^ Hospitals that did not report using a strategy at baseline but did report using it at follow-up were considered to have taken up that strategy.

We measured the uptake of each strategy separately, and we also created a count variable (possible range of 0–9) as the sum of strategies that were taken up. Our empirical data suggested a possible threshold effect with three strategies taken up; thus, we also dichotomized the count variable at zero to two versus three or more strategies taken up. We were unable to examine the association between RSRR change and uptake of several of the strategies that had previously been found to be either positively or negatively associated with RSRR in cross-sectional analysis,^8^ because not enough hospitals took up these strategies between the baseline and follow-up periods. Our longitudinal design could only analyze strategies whose use changed appreciably, which we operationalized as statistically significant increases within our sample.

We also obtained data on hospital characteristics from the 2009 American Hospital Association (AHA) annual survey. Variables included hospital size (total number of hospital beds), teaching status (COTH/non-COTH and teaching/non-teaching), ownership (for-profit/ nonprofit/government) and multihospital affiliation (yes/no). We determined census regions from the U.S. Census Bureau and ascertained area type (urban/suburban/rural) using the 2003 Urban Influence Codes. We also included a variable to indicate whether hospitals were members of H2H or STAAR.

### Statistical Analysis

We used standard frequency analyses to describe the sample of hospitals by key characteristics, and provided the weighted mean and standard error (SE) for hospital RSRR during 2010–2011 (baseline) and during 2011–2012 (follow-up). We described the baseline, follow-up, and changes in RSRR for hospitals by the number of strategies that hospitals had taken up between baseline and follow-up, and compared baseline and follow-up RSRR using a paired t-test. We used weighted linear regression to estimate unadjusted and adjusted associations between uptake of individual strategies and changes in RSRR, with follow-up RSRR as the outcome and the implementation of each strategy and baseline RSRR as independent variables. We estimated the association between the dummy variable (zero to two versus three or more strategies taken up) and follow-up RSRR, adjusted for baseline RSRR and hospital characteristics. We also tested whether the number of strategies taken up was associated with lower RSRR, adjusted for baseline RSRR and covariates, including the number of strategies (out of the nine listed in Text Box 1) in place at baseline. In all fully adjusted models, we included hospital characteristics and used a *p* value of < 0.05 as the threshold for statistical significance. Last, we examined whether participation in STAAR versus H2H modified the influence of the strategies taken up. We tested interaction terms one by one in fully adjusted models with appropriate main effects; given the large number of comparisons in this portion of the analysis, we used a *p* value of < 0.01 as the threshold for statistical significance. All analyses were weighted by hospital volume from 2011 to 2012, and because the frequency of missing data was low (< 3 %), we excluded cases with missing values. All analyses were completed with SAS 9.3 (Cary, NC).

## RESULTS

### Hospital Characteristics and RSRR

About half of the hospitals in our sample were teaching hospitals, with 85 % having at least 200 beds (Table 1). The overall RSRR at baseline (2010–2011) among the sample of hospitals was similar to the national median RSRR for patients with heart failure during this time (CMS, 2013). The mean RSRR across hospitals decreased from a baseline of 23.1 (SE =0.07; interquartile range (IQR) =1.8) to 22.3 (SE =0.08; IQR =2.2) at follow-up (*p* value for paired t-test < 0.001). Overall, 67 % of hospitals experienced an absolute reduction in RSRR, and change ranged from −5.47 to +5.59 between baseline and follow-up.

**Table 1.** Hospital Characteristics, Weighted by Hospital Volume (*N* = 478, Unweighted)

	%
Hospital teaching status	
Council of Teaching Hospitals member	21.3 %
Has accredited residency training	27.6 %
Non-teaching	51.2 %
Number of staffed beds	
< 200 beds	14.4 %
200–399 beds	38.6 %
400–599 beds	25.3 %
600+ beds	21.8 %
Census region	
New England	6.0 %
Middle Atlantic	13.1 %
East North Central	27.0 %
West North Central	6.8 %
South Atlantic	20.5 %
East South Central	9.6 %
West South Central	6.6 %
Mountain	2.5 %
Pacific	7.9 %
Geographic location	
Urban	91.9 %
Suburban	5.1 %
Rural	3.1 %
Ownership type	
For-profit	12.5 %
Nonprofit	80.2 %
Government	7.4 %
Multi-hospital affiliation	
Yes	69.8 %
No	30.2 %
Membership in initiatives to reduce readmissions	
STAAR	10.1 %
H2H	89.9 %
Number of strategies taken up	
0–2 strategies	74.7 %
3 or more strategies	25.3 %
30-day risk standardized readmission rate (RSRR)*	
Baseline (2010–2011); Mean [Range]	23.1 [18.4–30.0]
Follow-up (2011–2012); Mean [Range]	22.3 [17.6–27.3]
Change from baseline to follow-up; Mean [Range]	−0.76 [−5.5–5.6]

*Baseline and follow-up RSRR are significantly different (paired t-test = −9.01, *p* value < 0.001)

### Hospital Strategies and RSRR: Main Effects

The average number of strategies taken up from baseline to follow-up was 1.6 (SE =0.06); 22.2 % of hospitals took up no additional strategies during this time (Table 2). One-quarter (25.3 %) of hospitals took up at least three new strategies. Hospitals that took up three or more strategies had a reduction of 1.29 percentage points in RSRR (SE =0.17), whereas hospitals that took up zero to two strategies had a reduction of 0.57 percentage points in RSRR (SE =0.10).

**Table 2.** Weighted Distributions of Number of Strategies Taken Up by Hospitals and RSRR (*N* = 478)

Number of strategies^‡^	Unweighted N	%	Baseline RSRR (2010–2011) Mean	Follow-up RSRR (2011–2012) Mean	Change in RSRR Mean	Change in RSRR Mean (0-2 vs 3+ strategies)*
0	108	22.2	23.1	22.4	−0.68	−0.57
1	154	33.0	22.8	22.2	−0.54	
2	99	19.5	23.0	22.5	−0.51	
3	66	14.0	23.4	22.0	−1.35	−1.29
4	37	9.0	23.7	22.6	−1.14	
5	10	1.8	23.5	22.0	−1.57	
6–7^†^	4	0.5	24.9	23.3	−1.56	

*The difference in RSRR for hospitals with zero to two strategies versus hospitals with three or more strategies is statistically significant, *p* value < 0.01

^†^A single hospital took up seven strategies

^‡^We did not find statistically significant differences in follow-up RSRR adjusted for baseline RSRR for uptake of one additional strategy at any level other than two to three strategies

In both unadjusted and adjusted models examining the uptake of individual strategies, hospitals that adopted the strategy of routinely discharging patients with a follow-up appointment already scheduled experienced significant reductions in RSRR (Table 3). In the adjusted model, being in STAAR was associated with a significant relative increase in RSRR compared to being in H2H, as was adoption of the teach-back technique, which was a central recommendation of STAAR (Table 3); smaller hospital size (< 600 beds) was also associated with a relative increase in RSRR from baseline to follow-up.

**Table 3.** Linear Regression Models with Individual Strategies^1^ that Significantly Changed in Use Over the Study Period and Were Associated with Follow-Up RSRR, Adjusted for Baseline RSRR and Weighted by Hospital Volume

	Estimate (95 % CI)	
	Adjusted for Baseline RSRR only	Adjusted^‡, §, ll^ for All Variables
Patients are usually or always discharged from the hospital with an outpatient follow-up appointment already arranged	−0.53 (−0.93, −0.13)^†^	−0.63 (−1.03, −0.23)†
Hospital has partnered with other local hospitals to reduce readmission rates	0.31 (−0.09, 0.71)	0.37 (−0.03, 0.77)
Patients are regularly called after discharge to either follow-up on post-discharge needs or to provide additional education	−0.20 (−0.60, 0.19)	−0.13 (−0.53, 0.26)
Hospital tracks the following for quality improvement efforts		
Percent of patients discharged with follow-up appointment ≤ 7 days	−0.29 (−0.64, 0.07)	−0.17 (−0.54, 0.19)
Proportion of patients readmitted to another hospital	−0.23 (−0.68, 0.21)	−0.24 (−0.68, 0.21)
Estimates risk of readmission in a formal way and uses it in clinical care during patient hospitalization	−0.22 (−0.57, 0.13)	−0.29 (−0.65, 0.07)
Electronic medical record/web-based forms in place to facilitate medication reconciliation	−0.14 (−0.58, 0.30)	−0.31 (−0.74, 0.12)
At discharge, all heart failure patients (or their caregivers) receive written action plan for managing changes in condition	−0.08 (−0.44, 0.28)	0.06 (−0.30, 0.43)
Hospital promotes use of teach-back techniques for patient and family education	0.35 (−0.03, 0.73)	0.44 (0.07, 0.82)*
STAAR hospital (versus H2H)	0.44 (−0.05, 0.93)	0.72 (0.17, 1.27)†
Hospital teaching status		
Council of Teaching Hospitals member	−0.11 (−0.49, 0.27)	0.07 (−0.44, 0.57)
Has accredited residency training	−0.26 (−0.61, 0.09)	−0.18 (−0.56, 0.21)
Non-teaching	REF	REF
Number of staffed beds		
< 200 beds	0.60 (0.11, 1.10)*	0.79 (0.18, 1.41)*
200–399 beds	0.41 (0.01, 0.80)*	0.57 (0.11, 1.02)*
400–599 beds	0.40 (−0.03, 0.82)	0.67 (0.23, 1.11)†
600+ beds	REF	REF
Census region		
New England	−0.26 (−0.99, 0.46)	−0.66 (−1.41, 0.09)
Middle Atlantic	REF	REF
East North Central	0.09 (−0.40, 0.58)	−0.24 (−0.76, 0.27)
West North Central	−0.71 (−1.41, −0.02)*	−1.05 (−1.75, −0.34)†
South Atlantic	−0.19 (−0.71, 0.33)	−0.48 (−1.04, 0.08)
East South Central	−0.29 (−0.91, 0.33)	−0.63 (−1.32, 0.06)
West South Central	−0.00 (−0.70, 0.70)	−0.57 (−1.31, 0.17)
Mountain	−0.94 (−1.95, 0.08)	−1.32 (−2.32, −0.31)†
Pacific	−0.02 (−0.69, 0.64)	−0.59 (−1.29, 0.11)
Geographic location		
Urban	REF	REF
Suburban	−0.04 (−0.71, 0.64)	−0.33 (−1.01, 0.36)
Rural	−0.33 (−1.19, 0.53)	−0.41 (−1.29, 0.47)
Ownership type		
For-profit	REF	REF
Nonprofit	−0.58 (−1.03, −0.13)*	−0.40 (−0.92, 0.13)
Government	−0.21 (−0.89, 0.47)	0.36 (−0.43, 1.15)
Multi-hospital affiliation	0.32 (−0.00, 0.64)	0.48 (0.12, 0.84)^†^
Baseline RSRR (2010–2011)	0.50 (0.40, 0.59)†	0.45 (0.36, 0.55)^†^
R-Squared / Adjusted R-Squared		0.30 / 0.25

*p value < 0.05

^†^ *p* value < 0.01

^‡^Adjusted model uses data from 475 hospitals due to missing variables in three observations

^§^Adjusted model includes all strategies simultaneously and hospital characteristics [i.e., hospital membership in initiatives to reduce readmissions (H2H versus STAAR), teaching status, number of staffed beds, census region, geographic location, ownership type, multihospital affiliation]

^ll^All interactions with STAAR were nonsignificant except one. Among H2H hospitals, receiving an action plan at discharge was nonsignificant (*p* value = 0.57), whereas among STAAR hospitals, receiving an action plan at discharge was associated with increased RSRR (Estimate = 1.75; 95 % CI = 0.64, 2.87; *p* value = 0.002)

In both unadjusted and adjusted analyses of the number of strategies taken up, hospitals that took up three or more strategies had significantly greater reductions in RSRR compared with hospitals that took up only zero to two strategies (*p* values < 0.05) (Table 4). Hospitals that took up zero to two strategies had significantly lower average RSRR at baseline (22.9 %) compared with hospitals that took up three or more (23.5 %) (*p* value = 0.001) and had equivalent RSRR at follow-up (22.3 %). The association between three or more strategies taken up and greater reductions in RSRR was apparent both for hospitals that had below-median RSRR at baseline and for hospitals that had above-median RSRR at baseline (Table 4). In separate analyses, the number of strategies taken up as a continuous variable was not significant in unadjusted or adjusted analyses. Among the 117 hospitals (25.3 %) that took up three or more strategies, we found 93 unique combinations of strategies that were taken up.

**Table 4.** Linear Regression Models of Strategies Associated with Follow-up RSRR, Adjusted for Baseline RSRR and Weighted by Hospital Volume

	Estimate (95 % CI)^#^		
	Adjusted^‡, §^ Overall sample (*N* = 475)	Below Median Baseline RSRR of Sample (≤ 23 %) (*N* = 236)	Above Median Baseline RSRR of Sample (> 23 %) (*N* = 239)
Uptake			
0–2 strategies	REF	REF	REF
3 or more strategies^ll, ¶, **^	−0.40 (−0.74, −0.06)*	−0.53 (−1.07, −0.00)*	−0.47 (−0.92, −0.02)*
STAAR hospital (vs H2H)	0.67 (0.13, 1.21)*	0.38 (−0.43, 1.19)	1.06 (0.33, 1.79)^†^
Hospital teaching status			
Council of Teaching Hospitals member	−0.01 (−0.52, 0.49)	0.29 (−0.42, 1.01)	−0.53 (−1.25, 0.18)
Has accredited residency training	−0.18 (−0.56, 0.20)	−0.26 (−0.79, 0.27)	−0.18 (−0.73, 0.38)
Non-teaching	REF	REF	REF
Number of staffed beds			
< 200 beds	0.56 (−0.05, 1.18)	1.76 (0.91, 2.61)^†^	−0.53 (−1.43, 0.37)
200–399 beds	0.46 (−0.00, 0.91)*	1.68 (1.03, 2.33)^†^	−0.56 (−1.20, 0.09)
400–599 beds	0.46 (0.02, 0.90)*	1.26 (0.63, 1.90)^†^	−0.33 (−0.99, 0.32)
600+ beds	REF	REF	REF
Census region			
New England	−0.70 (−1.45, 0.06)	−0.52 (−1.63, 0.60)	−0.89 (−1.91, 0.13)
Middle Atlantic	REF	REF	REF
East North Central	−0.26 (−0.78, 0.26)	−0.49 (−1.33, 0.34)	−0.24 (−0.91, 0.44)
West North Central	−0.95 (−1.66, −0.25)^†^	−1.13 (−2.09, −0.16)*	−0.09 (−1.24, 1.06)
South Atlantic	−0.52 (−1.08, 0.04)	−0.16 (−1.02, 0.70)	−0.76 (−1.51, −0.01)*
East South Central	−0.71 (−1.40, −0.01)*	−0.95 (−2.02, 0.13)	−0.70 (−1.64, 0.23)
West South Central	−0.61 (−1.36, 0.13)	−0.55 (−1.65, 0.56)	−0.60 (−1.62, 0.43)
Mountain	−1.35 (−2.36, −0.33)^†^	−1.27 (−2.64, 0.09)	−1.34 (−2.83, 0.14)
Pacific	−0.57 (−1.28, 0.14)	−0.34 (−1.31, 0.62)	−1.21 (−2.44, 0.01)
Geographic location			
Urban	REF	REF	REF
Suburban	−0.28 (−0.98, 0.41)	−0.36 (−1.28, 0.56)	0.17 (−0.88, 1.23)
Rural	−0.34 (−1.22, 0.54)	−0.42 (−1.58, 0.74)	−0.61 (−2.00, 0.78)
Ownership type			
For-profit	REF	REF	REF
Nonprofit	−0.52 (−1.04, −0.00)*	−0.45 (−1.21, 0.30)	−0.42 (−1.12, 0.29)
Government	0.29 (−0.49, 1.08)	0.40 (−0.71, 1.51)	0.10 (−1.02, 1.22)
Multihospital affiliation	0.42 (0.06, 0.79)*	0.41 (−0.14, 0.96)	0.56 (0.08, 1.04)*
Baseline RSRR (2010–2011)	0.48 (0.38, 0.57)^†^	0.20 (−0.03, 0.44)	0.37 (0.18, 0.57)^†^
R-Squared / Adjusted R-Squared	0.27 / 0.23	0.26 / 0.18	0.18 / 0.10

*p value < 0.05

^†^ *p* value < 0.01

^‡^Adjusted model uses data from 475 hospitals due to missing variables in three observations

^§^Interaction with STAAR was nonsignificant

^ll^Unadjusted association Estimate = 0.41; 95 % CI = −0.76, −0.07; *p* value = 0.019

^¶^In separate models, the number of strategies taken up was not significantly associated with follow-up RSRR adjusted for baseline RSRR

^#^All analyses use RSRR as a continuous variable

**The number of strategies in place at baseline was not significant in the unadjusted or adjusted models and does not meaningfully alter the results

## DISCUSSION

Many hospitals that participated in H2H and STAAR initiatives to reduce unplanned readmissions between 2010 and 2012 had success. Among these hospitals, RSRR for patients with heart failure decreased from an average of 23.1 in 2010–2011 to 22.3 in 2011–2012. With more than 1 million Medicare admissions for heart failure per year, this reduction in RSRR is equivalent to the avoidance of hospitalization for thousands of patients per year.

The uptake of only one specific strategy was significantly associated with reductions in RSRR. Hospitals that took up the strategy of usually or always discharging patients with an outpatient follow-up appointment already scheduled experienced greater reduction in RSRR compared with hospitals that did not take up this strategy. This finding was consistent with earlier cross-sectional studies that have found the scheduling of follow-up appointments before discharge to be associated with lower RSRR,^8^^,^ 11 although this has not been examined independently in an experimental design. Being part of STAAR (compared with H2H) and taking up teach-back for patient and family education were both associated with higher RSRR adjusted for baseline RSRR; these effects may be due to unmeasured geographical variation, as the STAAR sample, in which teach-back was most prominent, was implemented in only three states.

We observed substantial hospital-level variation in improvement in RSRR, which provided an opportunity to understand what might distinguish hospitals in terms of strategies employed. Our results indicate that hospitals that took up three or more strategies experienced significantly greater reductions in RSRR. This finding is unlikely to be explained by ceiling or floor effects, as it was apparent both among hospitals that had below median RSRR at baseline and among hospitals that had above median RSRR at baseline. The number of baseline strategies in place was also not significantly different (*p* value = 0.178) between hospitals with below median RSRR and those with above median RSRR at baseline. The fact that hospitals that took up three or more strategies had higher average RSRR at baseline than hospitals that took up zero to two strategies suggests that hospitals with higher baseline RSRR may have been more motivated to try to reduce their readmissions.

Because so many different combinations of strategies were employed, we were limited in testing the relationship of any single set of strategies with changes in RSRR. The finding that hospitals with greater reductions in RSRR used a diversity of combinations of strategies highlights the complexity of interventions likely to be effective and may help explain the inconsistency of the literature concerning individual strategies. Despite a vast number of controlled trials and observational studies,^9^^–^ 11 ^,^ 18 ^,^ 15 ^,^ 24 ^,^ 17 ^,^ 25 ^,^ 26 ^,^ 13 ^,^ 16 ^,^ 14 ^,^ 27 ^,^ 28 empirical support for any single strategy is not uniform across studies.

Most striking was that for the 117 hospitals that took up three or more strategies in the present study and achieved greater reductions in RSRR, we found that 93 different combinations of strategies were employed. Several interpretations are possible. Hospitals that took up three or more additional strategies may have generally had more resources, which may have contributed to their improvement. Additionally, hospitals that took up three or more strategies may have implemented their strategies more effectively due to greater experience with employing improvement strategies, which has been shown to improve performance,^29^ or greater organizational attention to the issue. The narrower focus of H2H on cardiovascular diagnoses, compared with the broader reach of STAAR, may explain why H2H participants showed greater reductions in heart failure RSRR relative to STAAR participants. In addition, STAAR took place in only three states, so regional differences may have played a role. Lastly, our findings suggest that different sets of strategies may be effective for different organizational contexts. In this case, determining which strategies may be most impactful in differing organizational environments would be important for large-scale improvement.

Our findings should be interpreted in light of several limitations. First, the follow-up time in this study was relatively short (2010–2012), particularly as hospital strategies can be multifaceted and take time to implement consistently and to affect patterns of care. Over this relatively limited time period, the use of strategies that were previously found to be associated with lower RSRR^8^ in cross-sectional data had not changed significantly in their use, limiting our ability to fully examine their impact on changes in RSRR. The absence of a significant increase in collaboration with post-discharge healthcare providers and other community resources represents a particular gap. Future studies with longer follow-up periods are warranted. Second, our hospital sample is drawn from a selected group that participated in initiatives to reduce readmissions, and results in other hospitals may differ. Although the H2H and STAAR hospitals that we studied had baseline RSRRs similar to the national median for heart failure, hospitals that participated in these initiatives may have been more committed to devoting organizational resources to reducing readmission rates, and results in other hospitals may differ. Additionally, we examined readmission of patients with heart failure; findings may differ for other conditions. Third, given the limited prevalence of any single set of strategies taken up, we lacked statistical power to detect significant differences among sets of strategies, and few single strategies emerged as having a significant association with changes in RSRR. Fourth, the survey may have had some respondent bias in which those hospitals that improved or implemented strategies were more likely to respond; however, the response rate to the follow-up survey was very high (83.6 %) and many hospitals reported little change; thus, we do not think non-response is a large source of bias. Finally, hospital reports of their implementation of strategies may not always be accurate, due to misreporting and because the intensity and scope of the implementation may vary substantially. Further examination using qualitative methods would provide a more nuanced understanding of both strategy implementation and the resulting patterns of hospital performance improvement.

In conclusion, we found that many hospitals reduced readmission rates significantly over a 12–18 month period. About one-quarter of hospitals took up at least three new strategies, and these institutions achieved significantly greater reductions in unplanned readmissions than those that took up fewer or no strategies. Hospitals with the greater improvement did not exhibit any specific formula in strategy selection and many alternative approaches appeared to be successful. Overall, the improvement is significant but of modest magnitude, and particularly given the important role of community factors in readmission rates,^30^ increased coordination with resources outside the hospital will likely be needed to achieve national goals.

## Electronic supplementary material

Below is the link to the electronic supplementary material.

ESM 1 (PDF 226 kb)
