# Supplementary material for: Hospital Strategy Uptake and Reductions in Unplanned Readmission Rates for Patients with Heart Failure: A Prospective Study
Source: J Gen Intern Med. 2014 Dec 19;30(5):605–11. doi: 10.1007/s11606-014-3105-5 (PMC4395590; doi:10.1007/s11606-014-3105-5)
Supplement: Supplementary file 1 — (PDF 226 kb) [file 11606_2014_3105_MOESM1_ESM.pdf]

Note: All questions refer to patients with acute myocardial infarction (AMI) or heart failure (HF).
Please reflect upon your hospital's quality improvement efforts that are **CURRENTLY** in place

STAAR Evaluation Survey Instructions

Follow-up Survey
Summer 2013

This survey typically takes about 20 minutes to complete. Please note the following:

- **Finish Later –** If unable to complete the survey in a single session, you may save your answers by clicking the "**Finish Later**" button located at the bottom of each page. You may return to your survey as many times as needed using your ID and password until you complete the survey.
- **Logging Out** - The survey will automatically log you out if left open and idle for more than 30 minutes. You will be required to log back in. Your answers on completed pages of the survey will be saved, but answers on the survey page left open will not be saved and will require re-entry. We suggest using the "Finish Later" button if you need to leave the survey idle for more than 30 minutes.
- **Submit Survey** - When you are satisfied that your survey is complete, click the "**Complete**" button located on the bottom of the last page. Once completed, you will not be able to return to your survey.
- **Discussion and Collaboration** – To help answer questions, please feel free to consult with others at your hospital.

If you would like to preview the survey questions before proceeding, click on "**Preview Survey**", located in the left column of the Survey Home page. **We are available to assist you at 203-737-6114 or email (Marcia. mulligan@yale.edu) with questions or difficulties.**

Thank you very much for your time and participation!

Note: All questions refer to patients with acute myocardial infarction (AMI) or heart failure (HF).
Please reflect upon your hospital's quality improvement efforts that are **CURRENTLY** in place

I. Organizational support and quality improvement (QI) efforts for reducing readmission rates

1. Reducing preventable readmissions is a written objective for your hospital.

☐ Strongly agree ☐ Agree ☐ Not sure ☐ Disagree ☐ Strongly disagree

2a. Does your hospital have any quality improvement teams devoted to reducing preventable readmissions for patients with heart failure (HF)?

☐ YES ☐ NO
If "No", please skip to #3a

2b. Please indicate who belongs to the quality improvement (QI) team devoted to reducing readmission rates among patients with HF:

	**QI team members focusing on readmission for patients with HF**		
	Yes	No	Don't know
a. Senior management of the hospital - HF			
b. Hospital governing board members - HF			
c. Physicians - HF			
d. Advanced practice nurses or physician assistants - HF			
e. Nurses - HF			
f. Pharmacists - HF			
g. Social workers and/or case managers - HF			
h. Quality improvement/Quality management staff - HF			
i. Patient or family representatives - HF			
j. Others, specify: ____________________			

3a. Does your hospital have any quality improvement teams devoted to reducing preventable readmissions for patients with acute myocardial infarction (AMI)?

☐ YES ☐ NO
If "No", please skip to #4

Note: All questions refer to patients with acute myocardial infarction (AMI) or heart failure (HF).
Please reflect upon your hospital's quality improvement efforts that are **CURRENTLY** in place

3b. Please indicate who belongs to the quality improvement (QI) team devoted to reducing readmission rates among patients with AMI:

	**QI team members focusing on readmission for patients with AMI**		
	**Yes**	**No**	**Don't know**
a. Senior management of the hospital - AMI			
b. Hospital governing board members - AMI			
c. Physicians - AMI			
d. Advanced practice nurses or physician assistants - AMI			
e. Nurses - AMI			
f. Pharmacists - AMI			
g. Social workers and/or case managers - AMI			
h. Quality improvement/Quality management staff - AMI			
i. Patient or family representatives - AMI			
j. Others, specify: ____________________			

II. Participation in readmission collaboratives or campaigns

4. Please indicate if your hospital participates in each of the following collaboratives or campaigns, and if so, to what extent.

4a. State Action on Avoidable Rehospitalizations/Institute for Healthcare Improvement (STAAR)/(IHI)

☐ Yes, regularly participates
☐ Yes, occasionally or seldom participates
☐ No, does not participate
☐ Don't know

4b. Interventions to reduce acute care transfers (INTERACT)

☐ Yes, regularly participates
☐ Yes, occasionally or seldom participates
☐ No, does not participate
☐ Don't know

4c. Centers for Medicare & Medicaid/Quality Improvement Organizations Care Transitions Project

☐ Yes, regularly participates
☐ Yes, occasionally or seldom participates
☐ No, does not participate
☐ Don't know

4d. Better Outcomes for Older Adults through Safe Transitions (BOOST)/Society for Hospitalist Medicine

☐ Yes, regularly participates
☐ Yes, occasionally or seldom participates
☐ No, does not participate
☐ Don't know

4e. Project Reengineered Discharge (RED)

☐ Yes, regularly participates
☐ Yes, occasionally or seldom participates
☐ No, does not participate
☐ Don't know

4f. Hospital-to-Home (H2H)

☐ Yes, regularly participates
☐ Yes, occasionally or seldom participates
☐ No, does not participate
☐ Don't know

4g. Care Transitions Intervention (Coleman)

☐ Yes, regularly participates
☐ Yes, occasionally or seldom participates
☐ No, does not participate
☐ Don't know

4h. Transitional Care Model (Naylor)

☐ Yes, regularly participates
☐ Yes, occasionally or seldom participates
☐ No, does not participate
☐ Don't know

4i. University Health Systems Consortium collaborative

☐ Yes, regularly participates
☐ Yes, occasionally or seldom participates
☐ No, does not participate
☐ Don't know

4j. State hospital association collaborative

- ☐ Yes, regularly participates
- ☐ Yes, occasionally or seldom participates
- ☐ No, does not participate
- ☐ Don't know

4k. Local or regional collaborative

- ☐ Yes, regularly participates
- ☐ Yes, occasionally or seldom participates
- ☐ No, does not participate
- ☐ Don't know

4l. Others (please specify) ____________________________

III. Systems to reduce readmissions

IN-HOSPITAL CARE

5. During a patient's hospitalization, is the risk of death estimated in any formal way and also used in clinical care?

☐ YES ☐ NO

5a. If Yes, how? ____

6. During a patient's hospitalization, is the risk of readmission estimated in any formal way and also used in clinical care?

☐ YES ☐ NO

6a. If Yes, how? ____

7. Does your hospital have a multidisciplinary team to manage the care of patients who are at high risk of readmission?

☐ YES ☐ NO

8. Does your hospital have a reliable process in place to identify patients with heart failure at the time they are admitted?

☐ YES ☐ NO

Note: All questions refer to patients with acute myocardial infarction (AMI) or heart failure (HF).
Please reflect upon your hospital's quality improvement efforts that are **CURRENTLY** in place

9. Does your hospital have a reliable process in place to identify patients with AMI at the time they are admitted?

☐ YES ☐ NO

10. What proportion of your patients with AMI has a cardiologist involved in their care?

☐ ALL ☐ MOST ☐ SOME ☐ NONE

11. What proportion of your patients with HF has a cardiologist involved in their care?

☐ ALL ☐ MOST ☐ SOME ☐ NONE

MEDICATION RECONCILIATION

12. How often does each of the following occur as part of the medication reconciliation process at your hospital?

12a. Emergency medicine staff obtains medication history

☐ ALWAYS ☐ USUALLY ☐ SOMETIMES ☐ NEVER

12b. Admitting medical team obtains medication history

☐ ALWAYS ☐ USUALLY ☐ SOMETIMES ☐ NEVER

12c. Pharmacist or pharmacy technician obtains medication history

☐ ALWAYS ☐ USUALLY ☐ SOMETIMES ☐ NEVER

12d. Contact is made with outside pharmacies

☐ ALWAYS ☐ USUALLY ☐ SOMETIMES ☐ NEVER

12e. Contact is made with primary physician

☐ ALWAYS ☐ USUALLY ☐ SOMETIMES ☐ NEVER

12f. Outpatient and inpatient prescription records are linked electronically

☐ ALWAYS ☐ USUALLY ☐ SOMETIMES ☐ NEVER

12g. We subscribe to third party prescription database that provides historical fill and refill information (e.g., Health Care Systems)

☐ ALWAYS ☐ USUALLY ☐ SOMETIMES ☐ NEVER

13. What tools are in place to facilitate medication reconciliation at your hospital? **(Check all that apply)**

☐ Paper-based standardized form
☐ Web-based tool
☐ Form/tool built into electronic medical record
☐ No standardized form or tool is used for medication reconciliation
☐ Other, please specify____

14. Who is responsible for conducting medication reconciliation at discharge?

14a. Discharging physician

☐ ALWAYS ☐ USUALLY ☐ SOMETIMES ☐ NEVER

14b. Physician assistant or nurse practitioner

☐ ALWAYS ☐ USUALLY ☐ SOMETIMES ☐ NEVER

14c. Nurse

☐ ALWAYS ☐ USUALLY ☐ SOMETIMES ☐ NEVER

14d. Pharmacist

☐ ALWAYS ☐ USUALLY ☐ SOMETIMES ☐ NEVER

14e. Responsibility is not formally assigned

☐ ALWAYS ☐ USUALLY ☐ SOMETIMES ☐ NEVER

14f. Other (specify): _________________________

15. Is it a component of the discharge process to ask patients whether they can afford their medications?

☐ Yes, for all patients
☐ Yes, for some patients and/or for certain medications
☐ No, not routine

16. How often are your patients discharged from the hospital with their new medications in hand?

☐ ALWAYS ☐ USUALLY ☐ SOMETIMES ☐ NEVER

PATIENT/FAMILY EDUCATION

17. Does your hospital promote the use of teach-back techniques (having the patient "teach" new information back to the educator) for patient and family education?

☐ YES ☐ NO

18. What proportion of PATIENTS OR THEIR CAREGIVERS receives each of the following in written form at the time of discharge?

18a. Discharge instructions

☐ ALL ☐ MOST ☐ SOME ☐ NONE

18b. Discharge summary

☐ ALL ☐ MOST ☐ SOME ☐ NONE

18c. Educational information about heart failure, when relevant

☐ ALL ☐ MOST ☐ SOME ☐ NONE

18d. Educational information about AMI

☐ ALL ☐ MOST ☐ SOME ☐ NONE

18e. Action plan for patients with heart failure to help them manage changes in condition

☐ ALL ☐ MOST ☐ SOME ☐ NONE

18f. Personal health record (e.g., list of diagnoses, allergies, medications, physicians, contact information)

☐ ALL ☐ MOST ☐ SOME ☐ NONE

18g. Names, doses, and frequency of all discharge medications

☐ ALL ☐ MOST ☐ SOME ☐ NONE

18h. The purpose of each medication

☐ ALL ☐ MOST ☐ SOME ☐ NONE

18i. Information about which medications are new

☐ ALL ☐ MOST ☐ SOME ☐ NONE

18j. Information about which medications are have changed in dose or frequency

☐ ALL ☐ MOST ☐ SOME ☐ NONE

18k. Information about which medications are to be stopped

☐ ALL ☐ MOST ☐ SOME ☐ NONE

18l. The signs or symptoms that should prompt and immediate call to a physician or a return to the hospital

☐ ALL ☐ MOST ☐ SOME ☐ NONE

18m. Direct contact information for a specific physician to contact in case of emergency

☐ ALL ☐ MOST ☐ SOME ☐ NONE

18n. Any other type of emergency plan

☐ ALL ☐ MOST ☐ SOME ☐ NONE

18o. Side effects of each medication

☐ ALL ☐ MOST ☐ SOME ☐ NONE

19. Are all patients screened by a case manager using explicit criteria to identify post-discharge needs?

☐ YES ☐ NO

20. On the day of discharge, do patients leave the hospital with an outpatient follow-up appointment already arranged?

☐ ALWAYS ☐ USUALLY ☐ SOMETIMES ☐ NEVER

Note: All questions refer to patients with acute myocardial infarction (AMI) or heart failure (HF).
Please reflect upon your hospital's quality improvement efforts that are **CURRENTLY** in place

21. Is there a reliable process in place to ensure outpatient physicians are alerted to the patient's *admission* within 24 hours of admission?

☐ YES ☐ NO

22. Is there a reliable process in place to ensure outpatient physicians are alerted to the patient's *discharge* within 48 hours of discharge?

☐ YES ☐ NO

23. How quickly is a patient's discharge summary typically completed and available for viewing?

- ☐ On discharge
- ☐ Within 48 hours of discharge
- ☐ Within 7 days
- ☐ Within 30 days
- ☐ Longer than 30 days
- ☐ There are no explicit goals or policies defining a time-frame for completing the discharge summary

24. In what proportion of patients is a paper or electronic discharge summary sent directly to the patient's primary doctor?

☐ ALL ☐ MOST ☐ SOME ☐ NONE

25. What proportion of patients is cared for by outpatient physicians with access to inpatient electronic records?

☐ ALL ☐ MOST ☐ SOME ☐ NONE

26. Is there someone within the hospital assigned to follow up on test results that return after the patient is discharged?

☐ YES ☐ NO

27. Is there a process in place to ensure pending test results are listed in the discharge summary?

☐ YES ☐ NO

POST-ACUTE CARE LINKAGES AND SUPPORTS

28. Has your hospital partnered with community home care agencies and/or skilled nursing facilities to reduce readmission rates?

☐ YES ☐ NO

29. Has your hospital partnered with community physicians or physician groups to reduce readmission rates?

☐ YES ☐ NO

30. Has your hospital partnered with other local hospitals to reduce readmission rates?

☐ YES ☐ NO

31. Does your hospital regularly call patients after discharge to either follow up on post-discharge needs or to provide additional education?

☐ YES ☐ NO
If "No", please skip to #34

32. How long after discharge does your hospital regularly call patients?
(Check all that apply if multiple calls are made)

- ☐ Within 48 hours of discharge
- ☐ Within 1 week of discharge
- ☐ Within 2 weeks of discharge
- ☐ Within a month of discharge

33. Who conducts the calls? **(Check all that apply)**

- ☐ Clerical staff
- ☐ Care coordination/social work staff
- ☐ Nurses Pharmacist Physician
- ☐ Other, specify: ________________________

34. For how many of your patients does your hospital arrange home visits after discharge?

☐ ALL PATIENTS ☐ MOST ☐ SOME ☐ NONE

35. Does your hospital run its own post-discharge clinic in which patients can been seen within 7 days of discharge?

☐ YES ☐ NO

36. For how many of your patients does your hospital arrange telemonitoring after discharge?

☐ ALL PATIENTS ☐ MOST ☐ SOME ☐ NONE

37. How many of your patients with AMI does your hospital refer to cardiac rehabilitation after discharge?

☐ ALL PATIENTS ☐ MOST ☐ SOME ☐ NONE

38. How many of your patients does your hospital enroll in chronic care disease management programs after discharge?

☐ ALL PATIENTS ☐ MOST ☐ SOME ☐ NONE

39. Is there a physician assigned to coordinate with visiting nurse agencies about recently discharged patients in the post-discharge period?

☐ YES ☐ NO

40. For patients discharged with home health services, does your hospital provide direct contact information for a specific inpatient physician to contact in case of questions?

☐ YES ☐ NO

For the following questions, please consider patients who are transferred to skilled nursing facilities:

41. Does your hospital conduct a nurse-to-nurse report prior to transfer?

☐ ALWAYS ☐ USUALLY ☐ SOMETIMES ☐ NEVER

42. Does your hospital send a completed discharge summary with the patient?

☐ ALWAYS ☐ USUALLY ☐ SOMETIMES ☐ NEVER

43. Does your hospital send a reconciled medication list with the patient?

☐ ALWAYS ☐ USUALLY ☐ SOMETIMES ☐ NEVER

44. Does your hospital send a medication administration record with the patient?

☐ ALWAYS ☐ USUALLY ☐ SOMETIMES ☐ NEVER

45. Does your hospital provide a direct contact number to reach the inpatient treating physician?

☐ ALWAYS ☐ USUALLY ☐ SOMETIMES ☐ NEVER

Note: All questions refer to patients with acute myocardial infarction (AMI) or heart failure (HF).
Please reflect upon your hospital's quality improvement efforts that are **CURRENTLY** in place

IV. Measures and tracking

46. Does your hospital have a designated person or group to review unplanned readmissions that occur within 30 days of the original discharge?

☐ YES ☐ NO
If "No", please skip to #48

47. How long after the unplanned readmission are cases *typically* reviewed?

- ☐ Within one week of the readmission
- ☐ Within one month of the readmission
- ☐ Within 3 months of the readmission
- ☐ Other (please specify) ____
- ☐ We do not have a set timeframe for reviewing readmissions

48. Which of the following does your hospital track for quality improvement efforts?

a. Timeliness of discharge summaries	☐ YES ☐ NO
b. Proportion of discharge summaries that are sent to primary physician	☐ YES ☐ NO
c. Percent of patients discharged with a follow-up appointment	☐ YES ☐ NO
d. Percent of patients discharged with a follow-up appointment within 7 days	☐ YES ☐ NO
Accuracy of medication reconciliation	☐ YES ☐ NO
f. Content of discharge instructions	☐ YES ☐ NO
g. 30-day readmission rate	☐ YES ☐ NO
h. Early (<7 day) readmission rate	☐ YES ☐ NO
i. Proportion of patients readmitted to another hospital	☐ YES ☐ NO
j. Other, please specify	☐ YES ☐ NO

49. Please indicate your primary role in the hospital, check all that apply:

- ☐ Quality improvement, quality management, quality assurance, performance management
- ☐ Case management/care coordination/social work/discharge planning
- ☐ Cardiology
- ☐ Other clinical role
- ☐ Other non-clinical role

50. We are trying to better understand your professional network. Please list the hospitals that you specifically interact with regularly about efforts to improve the quality of cardiology care.

**HOSPITAL NAME**	**CITY**	**STATE**	**For each hospital, please choose one of the following**		
			**You** have provided **them** with information or tools to improve cardiology care	**They** have provided **you** with information or tools to improve cardiology care	Both

51. What about STAAR has been most useful to your hospital's efforts to reduce readmission rates?

52. How could STAAR improve the impact of H2H on your hospital?

*Questions deleted from survey on 8.16.13 due to H2H participation.
